# Systematic literature review and meta-analysis on the epidemiology of propionic acidemia

**DOI:** 10.1186/s13023-018-0987-z

**Published:** 2019-02-13

**Authors:** Tímea Almási, Lin T. Guey, Christine Lukacs, Kata Csetneki, Zoltán Vokó, Tamás Zelei

**Affiliations:** 1 Syreon Research Institute, Mexikói str. 65/A, Budapest, H-1142 Hungary; 2 Moderna, Inc., Cambridge, MA USA; 3 Department of Health Policy & Health Economics, Eötvös Loránd University, Budapest, Hungary

**Keywords:** Inherited metabolic disorder, Propionic acidemia, Epidemiology, Systematic literature review, Meta-analysis, Newborn screening

## Abstract

Propionic acidemia (PA, OMIM #606054) is a serious, life-threatening, inherited, metabolic disorder caused by the deficiency of the mitochondrial enzyme propionyl-coenzyme A (CoA) carboxylase (EC 6.4.1.3). The primary objective of this study was to conduct a systematic literature review and meta-analysis on the epidemiology of PA. The literature search was performed covering Medline, Embase, Cochrane Database of Systematic Reviews, CRD Database, Academic Search Complete, CINAHL and PROSPERO databases. Websites of rare disease organizations were also searched for eligible studies. Of the 2338 identified records, 188 articles were assessed for eligibility in full text, 43 articles reported on disease epidemiology, and 31 studies were included into the quantitative synthesis. Due to the rarity of PA, broadly targeted population-based prevalence studies are not available. Nonetheless, implementation of newborn screening programs has allowed the estimation of the birth prevalence data of PA across multiple geographic regions. The pooled point estimates indicated detection rates of 0.29; 0.33; 0.33 and 4.24 in the Asia-Pacific, Europe, North America and the Middle East and North Africa (MENA) regions, respectively. Our systematic literature review and meta-analysis confirm that PA is an ultra-rare disorder, with similar detection rates across all regions with the exception of the MENA region where the disease, similar to other inherited metabolic disorders, is more frequent.

**Electronic supplementary material:**

The online version of this article (10.1186/s13023-018-0987-z) contains supplementary material, which is available to authorized users.

## Background

Propionic acidemia (PA) (Online Mendelian Inheritance in Man (OMIM) number #606054) is a serious, life-threatening, inherited, metabolic disorder caused by the deficiency of the mitochondrial enzyme propionyl-coenzyme A (CoA) carboxylase (EC 6.4.1.3), which results in the accumulation of toxic metabolites such as propionic acid and 2-methylcitrate [1, 2]. The onset of PA occurs most frequently in the neonatal period, but also has a rarer late onset form [3]. The clinical manifestations include episodic life-threatening metabolic decompensations, growth impairment, movement disorders, seizures, basal ganglia lesions, pancreatitis, and cardiomyopathy [4]. The disease can lead to severe intellectual disability (IQ < 70) and speech delay, such that the majority of patients with PA require special education [5, 6]. Prognosis of PA is generally poor; patients with severe disease forms may die in the newborn period or later due to metabolic decompensations, cardiac complications (cardiomyopathy, arrhythmias) or basal ganglia stroke [4, 7, 8]. Milder or asymptomatic disease forms also exist, in these cases the prognosis may be more favorable [9].

There are no approved therapies that address the underlying root cause of PA. Current management of the disorder is limited to strict dietary management, carnitine supplementation, antibiotics such as metronidazole to reduce propionate production by intestinal bacteria, and ammonia scavengers such as carglumic acid to control episodes of hyperammonemia [4, 10]. Liver transplant as an approach to increase enzyme activity is a potential treatment option for severely affected individuals [4, 10].

Newborn screening for PA is performed in the United States, Australia and in several European and Asian countries [11]. Early detection by newborn screening is an effective approach to identify late onset cases [12, 13] and has been associated with decreased short-term mortality in PA [12, 14], however the impact on the long-term clinical course of the disorder is less clear [12–14]. PA cases can be detected in the neonatal period using acylcarnitine analysis by tandem mass spectrometry (MS/MS) on dried blood spots. Neonatal testing reveals elevated propionylcarnitine (C3) levels, and other secondary markers (methionine, C3/C2, and C3/C16 ratios) can be helpful to increase diagnostic accuracy [4]. Demonstration of deficient activity of propionyl-CoA carboxylase (PCC) or detection of pathogenic mutations in either PCCA (Mendelian Inheritance in Man (MIM) number 232000) or PCCB (MIM 232050) genes establishes the definitive diagnosis [10].

Although several studies reported results of newborn screening for PA in different regions, a systematic literature review on disease epidemiology has not been performed to date. The primary objective of this study was to conduct a systematic literature review and meta-analysis on the epidemiology of PA.

## Methods

### Systematic literature review

**Fig. 1:**
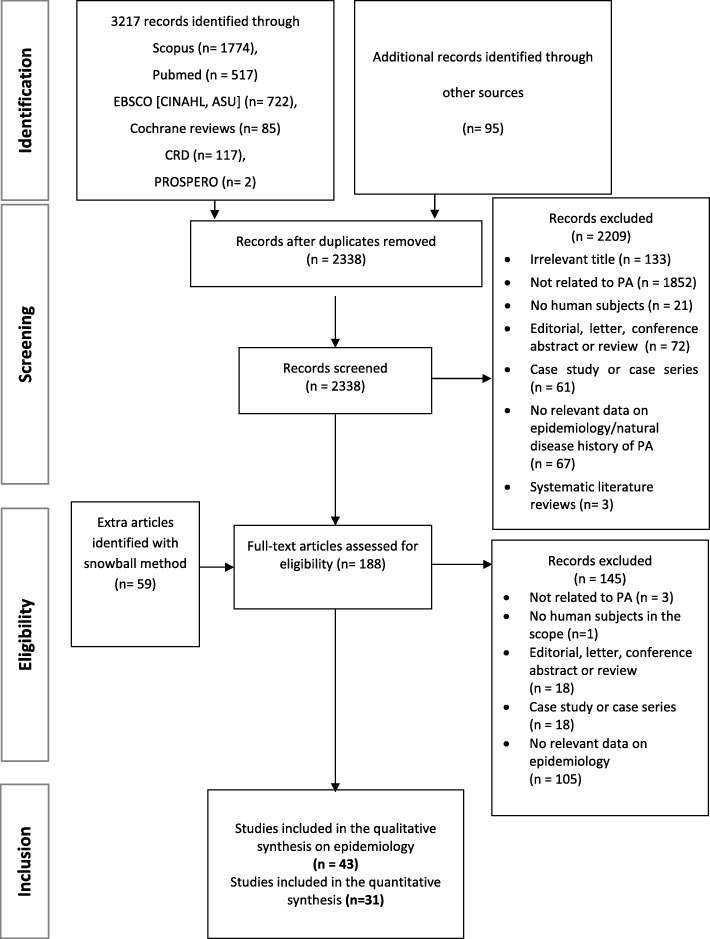
Flow of information diagram

The literature search was performed covering Medline, Embase, Cochrane Database of Systematic Reviews, Centre for Reviews and Dissemination (CRD) Database, Academic Search Complete, Cumulative Index to Nursing and Allied Health Literature (CINAHL) and PROSPERO databases. Websites of rare disease organizations were also searched for eligible studies. Detailed search strategies with the date of search and number of hits are summarized in Additional file 1: Table S1. The exclusion criteria of the title/abstract screening and full-text reviews are summarized in Fig. 1 and are detailed in Additional file 1: Table S2. A snowball method was also used to identify further relevant studies within the citations of full text papers.

Data extraction was performed by two independent researchers, and conflicts were resolved by discussion until a consensus was reached. During the full-text review, studies not reporting on a representative population for the given country or region were excluded. Reports on national screening programs with ~ 100% population coverage and analyses of national statistics were considered to provide the most accurate data on disease epidemiology. Reports on screening programs not covering ~ 100% of the population were considered to be eligible if a relatively large, random sample was used or the screening program had a multicenter design. Studies reporting on selected patient populations (e.g. patients with clinical suspicion of inborn error of metabolism) were excluded. Risk of bias was evaluated using the tool developed by Hoy et al. (2012) which is designed to assess methodological quality of prevalence studies [15].

### Meta-analysis

Studies with a high risk of bias were excluded from the quantitative synthesis. Overlap among the patient populations of multiple studies was rigorously investigated by reviewing countries, study periods, data sources and patient cohorts. Only the publication with the more complete dataset was included in the meta-analysis. A random effects meta-analysis was performed including all identified studies presenting birth prevalence, lifetime risk and cumulative incidence data. Heterogeneity between the individual study estimates was determined by the value of the heterogeneity chi-squared test and the I-square (I2) statistics. The metaprop module for STATA was used to perform all meta-analyses on STATA SE 15.0. This routine provides procedures for pooling proportions (in our case prevalence and cumulative incidence) in a meta-analysis of multiple studies. The confidence intervals of the individual study estimates are based on exact binomial (Clopper-Pearson) procedure [16]. Confidence intervals for the pooled estimates were calculated after Freeman-Tukey double arcsine transformation.

The analysis was performed separately for the following regions: North America, Europe, Asia-Pacific, Middle-East and North Africa. A time-specific subgroup analysis was also performed in order to observe the potential changes in disease occurrence throughout the years. The following two time periods were studied separately: 1981–2000 and 2001-present. A sensitivity analysis was also undertaken aiming to decrease the heterogeneity of epidemiological measures by omitting studies not presenting birth prevalence data.

## Results

After duplicates were removed, 2338 records were screened by their titles and abstracts from which 129 articles qualified for a full-text review. The snowball method identified 59 extra articles. In total, 188 articles were assessed for eligibility in full text and from these, 43 studies reported on the epidemiology of the disease (see Fig. 1). Among the 43 articles there were 11 overlapping studies and one using a different calculation method than the remaining articles, thus these 12 studies were further excluded from the quantitative analysis.

The largest share of publications originated from Europe, followed by the Asia-Pacific region. In the American continent, the United States was the most frequently investigated area, while in the Middle-East studies from Saudi Arabia were in the majority.

Large heterogeneity was observed regarding the epidemiological terms used in the identified papers. Therefore, the reported measures were recategorized based on their calculation methods according to the scientifically acceptable definitions of epidemiological terms (see Additional file 1: Table S3).

The vast majority of the articles reported on newborn screening programs providing estimates on the birth prevalence of the disease, defined as the number of affected newborns divided by the total population screened. Three articles followed a specific birth cohort over time and counted the number of diagnoses over the follow-up period, providing estimates on the cumulative incidence in the birth cohort [17–19]. In seven cases, authors divided the number of diagnosed patients by the number of live births during the same period of time, which measure aims to estimate the lifetime risk at birth [20]; a special case of cumulative incidence where the period of time studied is the entire remaining lifetime [21–27]. Although the calculation methods differ, the difference in the results is small if it is assumed that PA appears early in life, the disease occurrence is more or less constant, the size of birth cohorts and the diagnostic methods did not change significantly over time and all patients who have the underlying mutations will present with clinical symptoms over their lifetime. Based on these assumptions, we use the term “detection rate” for the three above-mentioned measures throughout the paper. Only one study calculated the proportion of affected patients within the total population providing the point prevalence of the disease [28]. Point prevalence is not comparable with the other frequency measures, therefore, this publication was excluded from the quantitative synthesis.

### Epidemiological data on PA – By territory

**Fig. 2:**
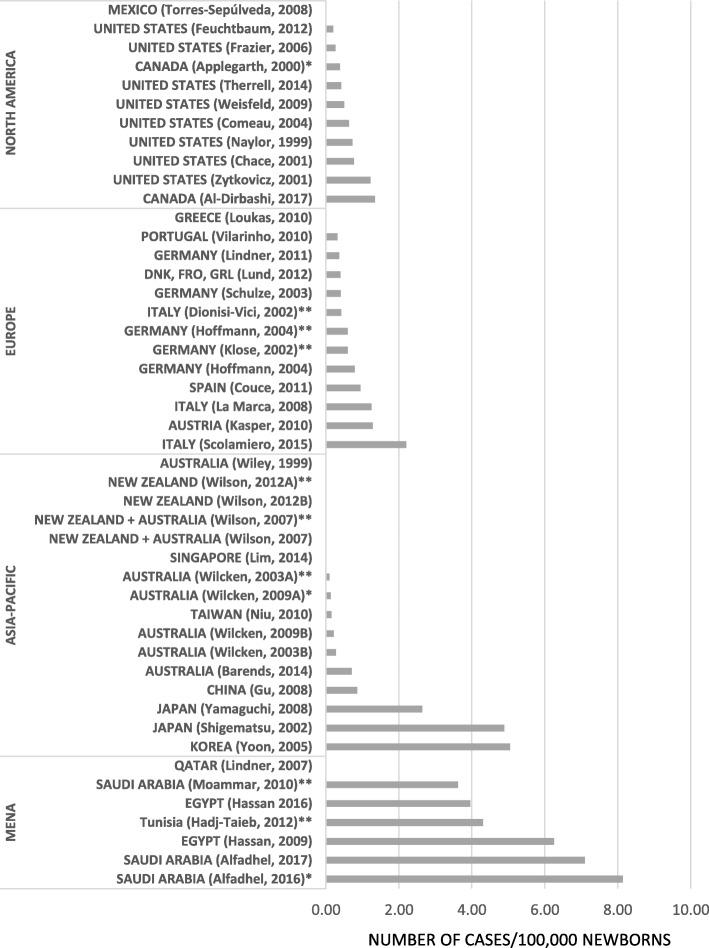
Estimates of birth prevalence of propionic acidemia in the different countries and geographical regions

In North America, detection rates of PA ranged between 0.20 (US, California) and 1.35 (Canada, Ontario) per 100,000 newborns [29, 30] (see Fig. 2) [17-19, 21-27, 29-42, 43-60]. The pooled point estimate indicated a detection rate of 0.33 per 100,000 newborns (CI: 0.11–0.63) in North America (see Table 1). All 8 articles originating from the US – except Zytkovicz et al. (2001) with 1.22 per 100,000 newborns – indicated a detection rate below 1 per 100,000 newborns. Subgroup analysis by time periods revealed some decrease in the detection rate between the periods of ‘1981–2000’ and ‘2001-present’; the detection rate decreased from 0.56 (CI: 0.23–1.01) to 0.26 (CI: 0.00–1.01) per 100,000 newborns, but the confidence intervals were largely overlapping.

**Table 1:** Base case and sensitivity analysis by geographic area (sensitivity analysis includes only studies with birth prevalence estimates derived from newborn screening studies) (detection rate per 100,000 newborns)

	Base case			Sensitivity analysis (birth prevalence)		
Continent	point estimate (95% CI)	Number of studies	I^2^	point estimate (95% CI)	Number of studies	I^2^
North America	0.33	6	33.76%	0.41	5	47.01%
	(0.11–0.63)			(0.09–0.89)		
Europe	0.33	10	23.83%	0.41	8	15.42%
	(0.15–0.57)			(0.13–0.80)		
Asia-Pacific	0.29	10(12 cohorts)	79.31%	0.48	9	79.68%
	(0.03–0.74)			(0.04–1.24)		
MENA	4.24	5	48.71%	4.48	3	28.73%
	(2.53–6.31)			(1.34–9.00)		

In Europe, the detection rates varied between 0.32 and 2.20 per 100,000 newborns [31, 32]. The pooled point estimate indicated a rate of 0.33 per 100,000 newborns (CI: 0.15–0.57) in Europe. Screening programs with 100% population coverage were identified in Austria, Italy, Spain and Portugal, where detection rates varied between 1.29 (Austria), 1.25 (Italy), 0.95 (Spain) and 0.32 (Portugal) per 100,000 newborns [32–35]. The largest reference population was identified in Italy, where the authors analyzed aggregate statistics from the period of 1985–1997 (*n* = 7,173,959 births) and found a detection rate of 0.42 per 100,000 newborns [21]. No time difference was shown by the subgroup analysis of the two time periods.

Detection rates in the region of Asia-Pacific were between 0.09 and 5.05 per 100,000 newborns [24, 36]. The conducted meta-analysis revealed a pooled point estimate of 0.29 per 100,000 newborns (CI: 0.03–0.74) for the Asia-Pacific region (see Table 1). The highest estimate from the region originates from Korea where Yoon et al. (2005) identified 4 cases among the 79,179 screened newborns (detection rate: 5.05/100,000 newborns) [36]. Relatively high occurrence characterized Japan where detection rates ranged between 2.64 and 4.89 per 100,000 newborns [37] [38]. Subgroup analysis by time periods indicated a possible increase in detection rate over the years, 0.08 (CI: 0.01–0.22) vs. 0.45 (CI: 0.02–1.25) per 100,000 newborns in the time periods of ‘1981–2000’ and ‘2001-present’.

Epidemiology studies performed in the Middle East and North Africa (MENA) region showed significantly increased detection rates compared to other regions. Out of the 8 identified articles, 6 reported detection rates over 3 per 100,000 newborns (range: 3.62 to 8.14 per 100,000 newborns) [17, 23, 27, 39–41]. The pooled point estimate was also relatively high, 4.24 per 100,000 newborns (CI: 2.53–6.31) (see Table 1) without considerable change over the years (4.11 (CI: 2.82–5.63) vs 4.48 (CI: 1.34–9.00) per 100,000 newborns in the time periods of ‘1981–2000’ and ‘2001-present’).

The only article that estimated (point) prevalence data originated from Oman where authors reported a prevalence of 0.40 per 100,000 inhabitants [28].

### Epidemiological data on PA subtypes and ethnicities

Only one study was identified which reported the proportion of PCCA-deficient and PCCB-deficient patients and was representative on a regional or country level [42], therefore no quantitative analysis could be conducted on the PA subtypes. The described newborn screening program identified 6 PA cases among the 847,418 screened newborns in Australia during 2002–2014. Gene analysis was conducted only on 3 PA patients, which resulted in 2 PCCA-deficient and 1 PCCB-deficient cases (detection rates of 0.24 and 0.12 per 100,000 newborns, respectively) [42].

Disease prevalence by ethnicities was investigated by Feuchtbaum et al. (2012) in the United States, in California [30]. Only Native Americans were characterized by a significantly higher detection rate (6.7 per 100,000 newborns) than the overall rate (0.2 per 100,000 newborns). Black and Hispanic ethnic groups showed detection rates of 0.8 and 0.3 patients per 100,000 newborns, respectively, but these differences did not reach statistical significant level. No PA cases were identified among other ethnicities.

## Discussion

Pooled point estimates of detection rates remained below 1 per 100,000 newborns in all regions, except the MENA where the results were significantly higher. This is in line with the findings of Chapman et al. (2018) who also identified higher birth prevalence in Kuwait than in the US or Southwest Germany; reported detection rates were 0.41 in the United States, 0.35 in Southwest Germany and 1.68 in Kuwait per 100,000 newborns [61]. Alfadhel et al. (2016 and 2017) explained the high numbers of metabolic disorders in Saudi Arabia by the frequent consanguineous marriages in the Saudi society [17, 39]. Al-Thihli et al. (2014) found that 95% of the investigated patients with inborn errors of metabolism (*n* = 229) were from consanguineous parents, while Moammar et al. (2010) detected a consanguineous rate of 100% among the affected patients [23, 28]. Epidemiology studies from Japan also reported higher detection rates ranging between 2.64 and 4.89 per 100,000 newborns. According to Yamaguchi et al. (2008); Shigematsu et al. (2002) and Yorifuji et al. (2003), the higher occurrence can be explained by a mutation (p. Y435C) in the PCCB gene that accounts for a mild form of PA [37, 38, 62]. Founder effects and thus higher detection rates of PA can also be found in such communities as the Amish and Mennonite communities [63], Galicians in Spain [33], and the Greenlandic Inuits in Greenland [64].

The epidemiological terminology used in the identified studies was heterogeneous and inconsistent. An added value of our study is a recategorization and harmonization of all published epidemiological measures (see Additional file 1: Table S3).

In most of meta-analyses performed, the I^2^ statistics indicated substantial heterogeneity across the studies that underlines the necessity of random effects meta-analysis. Subgroup analysis by two time periods did not reveal a substantial change in disease frequency throughout the years. Pooled point estimates remained under 1 per 100,000 newborns in both periods (‘1981–2000’ and ‘2001-present’) in all regions, except in the MENA, similar to the main analysis. Sensitivity analysis indicated that performing the meta-analyses using only birth prevalence data resulted in slightly higher estimates than the base case analysis. This might imply that newborn screening may result in a slight overestimation of the clinically relevant incidence since not all identified cases will necessarily develop clinical symptoms later on [22]. These patients without clinical symptoms might have a milder form of disease that may remain undiagnosed without systematic screening.

Due to the scarcity of studies with representative reference populations conducting PCCA- and PCCB-deficient subtype analysis, the current systematic literature review could not conclude on the relative detection rates of these subtypes. However, the distribution of PCCA-deficient and PCCB-deficient subtypes is reported to be approximately equally distributed [4] and no differences in severity or outcome have been described between the two subtypes.

Due to the rarity of PA, broadly targeted population-based prevalence studies are not available. However, reports on the results of newborn screening programs provided valuable, high quality data on the birth prevalence of the disease. Nonetheless, differences in case definitions and cut-off values, reference population size, the screening methods used and incomplete reporting may all have an influence on the number of identified and reported cases. In many cases the diagnostic tool and related cut-off values were not reported. In addition, studies did not always provide the rate of population coverage, which prevented the assessment of potential selection bias. Where screening programs reported on the number of false positive and negative findings, the number of positive cases were adjusted accordingly. However, follow-up time was not always long enough to assess adequately the screening performance. To summarize, a newborn screening that includes limited gene sequencing and applies appropriate follow-up can be the “gold standard” for measuring prevalence of most metabolic disorders and possibly non-metabolic genetic disorders as well.

Despite all the limitations mentioned above, our results indicated similar disease occurrence to the systematic literature review by the Spanish Health Technology Assessment Agency, conducted with the purpose of evaluating the clinical effectiveness of newborn screening programs [11]. Compared to this review, our research was not restricted to screening programs, therefore, it provides a more comprehensive overview on disease epidemiology.

## Conclusion

Implementation of newborn screening programs has allowed the estimation of the birth prevalence data of PA across multiple geographic regions. However, a certain evidence gap can be observed as epidemiological studies from South America, South Africa, Eastern Europe or Russia were not identified by our literature search. Our systematic literature review and meta-analysis confirm that PA is an ultra-rare disorder, with similar detection rates across all regions with the exception of the MENA region where the disease, similar to other inherited metabolic disorders, is more frequent.

## Additional file

Additional file 1: **Table S1.** Search strategies and number of hits in different databases. **Table S2.** Exclusion criteria during the title and abstract screening. **Table S3.** Definitions of epidemiological measures. (DOCX 20 kb)

## Abbreviations

CINAHL: The Cumulative Index to Nursing and Allied Health Literature
CRD Database: Centre for Reviews and Dissemination
MENA: Middle East and North Africa
MIM: Mendelian Inheritance in Man
MS/MS: Tandem Mass Spectometry
OMIM: Online Mendelian Inheritance in Man
PA: Propionic Acidemia
PCC: Propionyl-CoA carboxylase

## References

[1] Gilksman FJ (2018). Propionic Acidemia.

[2] Wongkittichote P, Mew NA, Chapman KA. Propionyl-CoA carboxylase–a review. Mol Genet Metab. 2017. 10.1016/j.ymgme.2017.10.002 PMC5725275 29033250

[3] Kölker S, Cazorla AG, Valayannopoulos V, Lund AM, Burlina AB, Sykut-Cegielska J (2015). The phenotypic spectrum of organic acidurias and urea cycle disorders. Part 1: the initial presentation. J Inherit Metab Dis.

[4] Shchelochkov OA, Carrillo N, Venditti C (2018). Propionic acidemia.

[5] De Baulny HO, Benoist J, Rigal O, Touati G, Rabier D, Saudubray J (2005). Methylmalonic and propionic acidaemias: management and outcome. J Inherit Metab Dis.

[6] Pena L, Burton BK (2012). Survey of health status and complications among propionic acidemia patients. Am J Med Genet A.

[7] Rousson R, Guibaud P (1984). Long term outcome of organic acidurias: survey of 105 French cases (1967–1983). J Inherit Metab Dis.

[8] Hamilton RL, Haas RH, Nyhan WL, Powell HC, Grafe MR (1995). Neuropathology of propionic acidemia: a report of two patients with basal ganglia lesions. J Child Neurol.

[9] Kuhara T, Ohse M, Inoue Y, Yorifuji T, Sakura N, Mitsubuchi H, et al. Gas chromatographic-mass spectrometric newborn screening for propionic acidaemia by targeting methylcitrate in dried filter-paper urine samples. J Inherit Metab Dis 2002 May;25(2):98–106. PubMed PMID: 12118533. Epub 2002/07/18. eng. 10.1023/a:1015620609075 12118533

[10] Baumgartner MR, Hörster F, Dionisi-Vici C, Haliloglu G, Karall D, Chapman KA (2014). Proposed guidelines for the diagnosis and management of methylmalonic and propionic acidemia. Orphanet J Rare Dis..

[11] Seoane Mato D, Cantero Muñoz, P., Atienza Merino, G. Clinical effectiveness of newborn screening for inborn errors of metabolism. Part II: Methylmalonic acidemia, Propionic acidemia, Tyrosinemia type I. Health Technology Assessment Database no. 4. 2014.

[12] Grünert S, Müllerleile S, De Silva L, Barth M, Walter M, Walter K (2012). Propionic acidemia: neonatal versus selective metabolic screening. J Inherit Metab Dis.

[13] Heringer J, Valayannopoulos V, Lund AM, Wijburg FA, Freisinger P, Barić I (2016). Impact of age at onset and newborn screening on outcome in organic acidurias. J Inherit Metab Dis.

[14] Dionisi-Vici C, Deodato F, Röschinger W, Rhead W, Wilcken B (2006). ‘Classical’organic acidurias, propionic aciduria, methylmalonic aciduria and isovaleric aciduria: long-term outcome and effects of expanded newborn screening using tandem mass spectrometry. J Inherit Metab Dis.

[15] Hoy D, Brooks P, Woolf A, Blyth F, March L, Bain C (2012). Assessing risk of bias in prevalence studies: modification of an existing tool and evidence of interrater agreement. J Clin Epidemiol.

[16] Newcombe RG (1998). Two-sided confidence intervals for the single proportion: comparison of seven methods. Stat Med.

[17] Alfadhel M, Benmeakel, M., Hossain, M. A., Al Mutairi, F., Al Othaim, A., Alfares, A. A., Al Balwi, M., Alzaben, A., Eyaid, W. Thirteen year retrospective review of the spectrum of inborn errors of metabolism presenting in a tertiary center in Saudi Arabia. Orphanet Journal of Rare Diseases. 2016;11(1). Pubmed Central PMCID: 2.7. 10.1186/s13023-016-0510-3 PMC5024448 27629047

[18] Applegarth DA, Toone JR. Incidence of inborn errors of metabolism in British Columbia, 1969–1996. Pediatrics. 2000;105(1):e10-e. 10.1542/peds.105.1.e10 10617747

[19] Wilcken B, Haas M, Joy P, Wiley V, Bowling F, Carpenter K, et al. Expanded newborn screening: outcome in screened and unscreened patients at age 6 years. Pediatrics 2009;124(2):e241–e248. PubMed PMID: 19620191. Epub 2009/07/22. eng. 10.1542/peds.2008-0586 19620191

[20] Foss AH, Duffner PK, Carter RL. Lifetime risk estimators in epidemiological studies of Krabbe Disease: Review and Monte Carlo comparison. Rare diseases (Austin, Tex). 2013;1:e25212. PubMed PMID: 25003000. Pubmed Central PMCID: PMC4070066. Epub 2013/01/01. eng. 10.4161/rdis.25212 PMC4070066 25003000

[21] Dionisi-Vici C, Rizzo C, Burlina AB, Caruso U, Sabetta G, Uziel G (2002). Inborn errors of metabolism in the Italian pediatric population: a national retrospective survey. J Pediatr.

[22] Hoffmann GF, von Kries R, Klose D, Lindner M, Schulze A, Muntau AC (2004). Frequencies of inherited organic acidurias and disorders of mitochondrial fatty acid transport and oxidation in Germany. Eur J Pediatr.

[23] Moammar H, Cheriyan G, Mathew R, Al-Sannaa N (2010). Incidence and patterns of inborn errors of metabolism in the Eastern Province of Saudi Arabia, 1983-2008. Annals of Saudi medicine.

[24] Wilcken B, Wiley V, Hammond J, Carpenter K (2003). Screening newborns for inborn errors of metabolism by tandem mass spectrometry. N Engl J Med.

[25] Wilson C, Kerruish, N. J., Wilcken, B., Wiltshire, E., Bendikson, K., Webster, D. Diagnosis of disorders of intermediary metabolism in New Zealand before and after expanded newborn screening: 2004–2009. New Zealand Medical Journal. 2012;125(1348):42–50. Pubmed Central PMCID: 2.7. 22282276

[26] Wilson C, Kerruish NJ, Wilcken B, Wiltshire E, Webster D. The failure to diagnose inborn errors of metabolism in New Zealand: the case for expanded newborn screening. The New Zealand Medical Journal (Online). 2007;120(1262). 17891215

[27] Hadj-Taieb S, Nasrallah F, Hammami M, Elasmi M, Sanhaji H, Moncef F (2012). Aminoacidopathies and organic acidurias in Tunisia: a retrospective survey over 23 years. La Tunisie Medicale.

[28] Al-Thihli K, Al-Murshedi, F., Al-Hashmi, N., Al-Mamari, W., Islam, M. M., Al-Yahyaee, S. A. Consanguinity, endogamy and inborn errors of metabolism in Oman: A cross-sectional study. Human Heredity. 2014;77(1–4):183–8. Pubmed Central PMCID: 2.7. 10.1159/000362686 25060282

[29] Al-Dirbashi OY, McIntosh N, Chakraborty P (2017). Quantification of 2-methylcitric acid in dried blood spots improves newborn screening for propionic and methylmalonic acidemias. J Med Screen.

[30] Feuchtbaum L, Carter J, Dowray S, Currier RJ, Lorey F. Birth prevalence of disorders detectable through newborn screening by race/ethnicity. Genet Med 2012;14(11):937–945. PubMed PMID: 22766612. Epub 2012/07/07. eng. 10.1038/gim.2012.76 22766612

[31] Scolamiero E, Cozzolino C, Albano L, Ansalone A, Caterino M, Corbo G (2015). Targeted metabolomics in the expanded newborn screening for inborn errors of metabolism. Mol BioSyst.

[32] Vilarinho L, Rocha H, Sousa C, Marcão A, Fonseca H, Bogas M (2010). Four years of expanded newborn screening in Portugal with tandem mass spectrometry. J Inherit Metab Dis.

[33] Couce L, Castiñeiras, Daisy E., Bóveda, Dolores, Baña, Ana, Cocho, José A., Iglesias, Agustín J., Colón, Cristobal, Alonso-Fernández, José R., Fraga, José M. Evaluation and long-term follow-up of infants with inborn errors of metabolism identified in an expanded screening programme. Molecular Genetics and Metabolism. 2011;104(4):470–5. PubMed PMID: 67514211. Pubmed Central PMCID: 2.7. 10.1016/j.ymgme.2011.09.021 22000754

[34] Kasper DC, Ratschmann, R., Metz, T. F., Mechtler, T. P., Möslinger, D., Konstantopoulou, V., Item, C. B., Pollak, A., Herkner, K. R. The National Austrian Newborn Screening Program - Eight years experience with mass spectrometry. Past, present, and future goals. Wiener Klinische Wochenschrift. 2010;122(21–22):607–13. Pubmed Central PMCID: 2.7. 10.1007/s00508-010-1457-3 20938748

[35] la Marca G, Malvagia S, Casetta B, Pasquini E, Donati MA, Zammarchi E (2008). Progress in expanded newborn screening for metabolic conditions by LC-MS/MS in Tuscany: update on methods to reduce false tests. J Inherit Metab Dis.

[36] Yoon H-R, Lee, Kyung Ryul, Kang, Seungwoo, Lee, Dong Hwan, Yoo, Han-Wook, Min, Won-Ki, Cho, Dong Hee, Shin, Son Moon, Kim, Jongwon, Song, Junghan, Yoon, Ho Joo, Seo, Sonsang, Hahn, Si Houn. Screening of newborns and high-risk group of children for inborn metabolic disorders using tandem mass spectrometry in South Korea: a three-year report. Clinica Chimica Acta. 2005;354(1/2):167–80. PubMed PMID: 17437245. Pubmed Central PMCID: 2.7. 10.1016/j.cccn.2004.11.032 15748614

[37] Yamaguchi S (2008). Newborn screening in Japan: restructuring for the new era. Ann Acad Med Singap.

[38] Shigematsu Y, Hirano S, Hata I, Tanaka Y, Sudo M, Sakura N, et al. Newborn mass screening and selective screening using electrospray tandem mass spectrometry in Japan. J Chromatogr B Anal Technol Biomed Life Sci 2002;776(1):39–48. PubMed PMID: 12127323. Epub 2002/07/20. eng. 10.1016/s1570-0232(02)00077-6 12127323

[39] Alfadhel M, Al Othaim A, Al Saif S, Al Mutairi F, Alsayed M, Rahbeeni Z (2017). Expanded newborn screening program in Saudi Arabia: incidence of screened disorders. J Paediatr Child Health.

[40] Hassan FA, El-Mougy F, Sharaf SA, Mandour I, Morgan MF, Selim LA (2016). Inborn errors of metabolism detectable by tandem mass spectrometry in Egypt: the first newborn screening pilot study. J Med Screen.

[41] Hassan Fayza FEM, Mandour I, Hady SA, Selim L, Atty SA, Morgan M, Salem F, Oraby A, Mehaney D, Monem MA, El Nekhely I (2009). Nadia Moharam preliminary results of Egypt experience for use of tandem mass spectrometry for expanded metabolic screening. J Appl Sci Res.

[42] Barends M, Pitt J, Morrissy S, Tzanakos N, Boneh A (2014). Biochemical and molecular characteristics of patients with organic acidaemias and urea cycle disorders identified through newborn screening. Mol Genet Metab.

[43] Therrell BL, Lloyd-Puryear, M. A., Camp, K. M., Mann, M. Y. Inborn errors of metabolism identified via newborn screening: Ten-year incidence data and costs of nutritional interventions for research agenda planning. Molecular Genetics and Metabolism. 2014;113(1):14–26. Pubmed Central PMCID: 2.7. 10.1016/j.ymgme.2014.07.009 PMC4177968 25085281

[44] Weisfeld-Adams JD, Morrissey MA, Kirmse BM, Salveson BR, Wasserstein MP, McGuire PJ, et al. Newborn screening and early biochemical follow-up in combined methylmalonic aciduria and homocystinuria, cblC type, and utility of methionine as a secondary screening analyte. Mol Genet Metab 2010;99(2):116–123. PubMed PMID: 19836982. Pubmed Central PMCID: PMC2914534. Epub 2009/10/20. eng. 10.1016/j.ymgme.2009.09.008 PMC2914534 19836982

[45] Wiley V, Carpenter K, Wilcken B. Newborn screening with tandem mass spectrometry: 12 months' experience in NSW Australia. Acta paediatrica (Oslo, Norway : 1992) Supplement. 1999 Dec;88(432):48–51. PubMed PMID: 10626578. Epub 2000/01/08. eng. 10.1111/j.1651-2227.1999.tb01157.x 10626578

[46] Zytkovicz TH, Fitzgerald EF, Marsden D, Larson CA, Shih VE, Johnson DM (2001). Tandem mass spectrometric analysis for amino, organic, and fatty acid disorders in newborn dried blood spots: a two-year summary from the New England newborn screening program. Clin Chem.

[47] Chapman KA, Gramer G, Viall S, Summar ML (2018). Incidence of maple syrup urine disease, propionic acidemia, and methylmalonic aciduria from newborn screening data. Molecular genetics and metabolism reports.

[48] Yorifuji T, Kawai, M., Muroi, J., Mamada, M., Kurokawa, K., Shigematsu, Y., Hirano, S., Sakura, N., Yoshida, I., Kuhara, T., Endo, F., Mitsubuchi, H., Nakahata, T. Erratum: Unexpectedly high prevalence of the mild form of propionic acidemia in Japan: Presence of a common mutation and possible clinical implications (Human Genetics (2002) 111 (161–165)). Human Genetics. 2003;112(1):100. Pubmed Central PMCID: 2.1. 10.1007/s00439-002-0761-z 12189489

[49] Puffenberger E, editor Genetic heritage of the Old Order Mennonites of southeastern Pennsylvania. American Journal of Medical Genetics Part C: Seminars in Medical Genetics; 2003: Wiley Online Library. 10.1002/ajmg.c.20003 12888983

[50] Ravn K, Chloupkova M, Christensen E, Brandt NJ, Simonsen H, Kraus JP (2000). High incidence of propionic acidemia in Greenland is due to a prevalent mutation, 1540insCCC, in the gene for the β-subunit of propionyl CoA carboxylase. Am J Hum Genet.

[51] Chace DH, DiPerna JC, Kalas TA, Johnson RW, Naylor EW (2001). Rapid diagnosis of methylmalonic and propionic acidemias: quantitative tandem mass spectrometric analysis of propionylcarnitine in filter-paper blood specimens obtained from newborns. Clin Chem.

[52] Comeau AM, Larson C, Eaton RB. Integration of new genetic diseases into statewide newborn screening: New England experience. Am J Med Genet C: Semin Med Genet 2004;125C(1):35–41. PubMed PMID: 14755432. Epub 2004/02/03. eng. 10.1002/ajmg.c.30001 14755432

[53] del Rosario T-SM, Martínez-de Villarreal LE, Esmer C, González-Alanís R, Ruiz-Herrera C, Sánchez-Peña A (2008). Tamiz metabólico neonatal por espectrometría de masas en tándem: dos años de experiencia en Nuevo León. México salud pública de méxico.

[54] Frazier D, Millington D, McCandless S, Koeberl D, Weavil S, Chaing S (2006). The tandem mass spectrometry newborn screening experience in North Carolina: 1997–2005. J Inherit Metab Dis.

[55] Gu X, Wang Z, Ye J, Han L, Qiu W (2008). Newborn screening in China: phenylketonuria, congenital hypothyroidism and expanded screening. Ann Acad Med Singap.

[56] Klose DA, Kolker S, Heinrich B, Prietsch V, Mayatepek E, von Kries R, et al. Incidence and short-term outcome of children with symptomatic presentation of organic acid and fatty acid oxidation disorders in Germany. Pediatrics 2002;110(6):1204–1211. PubMed PMID: 12456920. Epub 2002/11/29. eng. 10.1542/peds.110.6.1204 12456920

[57] Lim J, Tan E, John C, Poh S, Yeo S, Ang J (2014). Inborn error of metabolism (IEM) screening in Singapore by electrospray ionization-tandem mass spectrometry (ESI/MS/MS): an 8year journey from pilot to current program. Mol Genet Metab.

[58] Lindner M, Abdoh G, Fang-Hoffmann J, Shabeck N, Al Sayrafi M, Al Janahi M (2007). Implementation of extended neonatal screening and a metabolic unit in the State of Qatar: developing and optimizing strategies in cooperation with the neonatal screening Center in Heidelberg. J Inherit Metab Dis.

[59] Lindner M, Gramer G, Haege G, Fang-Hoffmann J, Schwab KO, Tacke U (2011). Efficacy and outcome of expanded newborn screening for metabolic diseases-report of 10 years from south-West Germany. Orphanet J Rare Dis.

[60] Loukas YL, Soumelas G-S, Dotsikas Y, Georgiou V, Molou E, Thodi G (2010). Expanded newborn screening in Greece: 30 months of experience. J Inherit Metab Dis.

[61] Lund AM, Hougaard, David Michael, Simonsen, Henrik, Andresen, Brage Storstein, Christensen, Mette, Dunø, Morten, Skogstrand, Kristin, Olsen, Rikke K. J., Jensen, Ulrich Glümer, Cohen, Arieh, Larsen, Nanna, Saugmann-Jensen, Peter, Gregersen, Niels, Brandt, Niels Jacob, Christensen, Ernst, Skovby, Flemming, Nørgaard-Pedersen, Bent. Biochemical screening of 504,049 newborns in Denmark, the Faroe Islands and Greenland — Experience and development of a routine program for expanded newborn screening. Molecular Genetics and Metabolism. 2012;107(3):281–93. PubMed PMID: 82897407. Pubmed Central PMCID: 2.7. 10.1016/j.ymgme.2012.06.006 22795865

[62] Naylor EW, Chace DH. Automated tandem mass spectrometry for mass newborn screening for disorders in fatty acid, organic acid, and amino acid metabolism. J Child Neurol 1999;14 Suppl 1:S4–S8. PubMed PMID: 10593560. Epub 1999/12/11. eng. 10.1177/0883073899014001021 10593560

[63] Niu D-M, Chien Y-H, Chiang C-C, Ho H-C, Hwu W-L, Kao S-M (2010). Nationwide survey of extended newborn screening by tandem mass spectrometry in Taiwan. J Inherit Metab Dis.

[64] Schulze A, Lindner M, Kohlmuller D, Olgemoller K, Mayatepek E, Hoffmann GF. Expanded newborn screening for inborn errors of metabolism by electrospray ionization-tandem mass spectrometry: results, outcome, and implications. Pediatrics 2003;111(6 Pt 1):1399–1406. PubMed PMID: 12777559. Epub 2003/06/05. eng. 10.1542/peds.111.6.1399 12777559

