# Supplementary material for: Systematic literature review and meta-analysis on the epidemiology of propionic acidemia
Source: Orphanet J Rare Dis. 2019 Feb 13;14:40. doi: 10.1186/s13023-018-0987-z (PMC6375193; doi:10.1186/s13023-018-0987-z)
Supplement: Supplementary file 1 — Table S1. Search strategies and number of hits in different databases. Table S2. Exclusion criteria during the title and abstract screening. Table S3. Definitions of epidemiological measures. (DOCX 20 kb) [file 13023_2018_987_MOESM1_ESM.docx]

Supplementary material

*Supplementary Table 1 Search strategies and number of hits in different databases*

| **Search No.** | **Search area** | **Search** | **Date of search** | **Number of hits** |
| --- | --- | --- | --- | --- |
| **Pubmed** (Medline) |  |  |  |  |
| #1 | Disease | ((( propion*[Title/Abstract] ) AND ( acidemia[Title/Abstract] OR acidaemia[Title/Abstract] OR aciduria ) ) OR ( ( ( ( propionyl[Title/Abstract] ) AND ( coa[Title/Abstract] OR "coenzyme a"[Title/Abstract] ) ) OR ( propionic[Title/Abstract] OR "PCCA"[Title/Abstract] OR "PCCB"[Title/Abstract] OR "PCC"[Title/Abstract] ) ) AND ( deficien*[Title/Abstract] OR disorder[Title/Abstract] OR defect*[Title/Abstract] OR insufficien*[Title/Abstract] ) OR ( propionicaciduria[Title/Abstract] OR propionicacidemia[Title/Abstract] OR propionicacidaemia[Title/Abstract] ) )) | 23/01/2018 | 2,106 |
| #2 | Clinical burden | (inciden*[Title/Abstract] OR prevalen*[Title/Abstract] OR demograph*[Title/Abstract] OR epidemiolog*[Title/Abstract] OR frequen*[Title/Abstract] OR rate[Title/Abstract] OR distribut*[Title/Abstract] OR "lifetime risk"[Title/Abstract] OR survival[Title/Abstract] OR mortality[Title/Abstract] OR outcome[Title/Abstract] OR "natural history"[Title/Abstract] OR "genetic heterogeneity"[Title/Abstract] OR progression[Title/Abstract]) | 23/01/2018 | 6,425,183 |
| #3 | Combined search – Clinical burden | #1 AND #2  Filters: English | 23/01/2018 | **517** |
| **SCOPUS** |  |  |  |  |
| #1 | Disease | TITLE-ABS-KEY ( propion* AND ( acidemia OR acidaemia OR aciduria ) ) OR TITLE-ABS-KEY ( ( ( propionyl AND ( coa OR "coenzyme a" ) ) OR propionic OR "PCCA" OR "PCCB" OR "PCC" ) AND ( deficien* OR disorder OR defect* OR insufficien* ) ) OR TITLE-ABS-KEY ( propionicaciduria OR propionicacidemia OR propionicacidaemia ) | 22/01/2018 | 5,424 |
| #2 | Clinical burden | (TITLE-ABS-KEY ( inciden* OR prevalen* OR demograph* OR epidemiolog* OR frequen* OR rate OR distribut* OR "lifetime risk" OR survival OR mortality OR outcome OR "natural history" OR "genetic heterogeneity" OR progression ) ) | 22/01/2018 | 17,838,389 |
| #3 | Combined search – Clinical burden | #1 AND #2 AND  ( LIMIT-TO ( LANGUAGE , "English" ) ) | 22/01/2018 | **1,774** |
| **EBSCO**  (CINAHL) |  |  |  |  |
| #1 | Disease | ( ( propion* AND ( acidemia OR acidaemia OR aciduria ) ) ) OR ( ( ( ( propionyl AND ( coa OR "coenzyme a" ) ) OR propionic OR "PCCA" OR "PCCB" OR "PCC" ) AND ( deficien* OR disorder OR defect* OR insufficien* ) ) ) OR ( ( propionicaciduria OR propionicacidemia OR propionicacidaemia ) ) | 22/01/2018 | 132 |
| #2 | Clinical burden | inciden* OR prevalen* OR demograph* OR epidemiolog* OR frequen* OR rate OR distribut* OR "lifetime risk" OR survival OR mortality OR outcome OR "natural history" OR “genetic heterogeneity” OR progression | 22/01/2018 | 1,014,818 |
| #3 | Combined search – Clinical burden | #1 AND #2 Narrow by Language: - English | 22/01/2018 | **79** |
| **EBSCO**  (Academic Search Ultimate) |  |  |  |  |
| #1 | Disease | ( ( propion* AND ( acidemia OR acidaemia OR aciduria ) ) ) OR ( ( ( ( propionyl AND ( coa OR "coenzyme a" ) ) OR propionic OR "PCCA" OR "PCCB" OR "PCC" ) AND ( deficien* OR disorder OR defect* OR insufficien* ) ) ) OR ( ( propionicaciduria OR propionicacidemia OR propionicacidaemia ) ) | 23/01/2018 | 1,855 |
| #2 | Clinical burden | inciden* OR prevalen* OR demograph* OR epidemiolog* OR frequen* OR rate OR distribut* OR "lifetime risk" OR survival OR mortality OR outcome OR "natural history" OR “genetic heterogeneity” OR progression | 23/01/2018 | 5,594,815 |
| #3 | Combined search – Clinical burden | #1 AND #2 Narrow by Language: - English | 23/01/2018 | **643** |
| **CRD Database** |  |  |  |  |
| #1 | Combined search | (propion* OR PCC)  AND  (prevalen* OR inciden* OR demograph* OR emidemiolog* OR frequen* OR rate OR distribut* OR lifetime risk OR survival OR mortality OR outcome OR history OR heterogeneity OR progression) | 22/01/2018 | **117** |
| **PORSPERO** |  |  |  |  |
| #1 | Search | propion* AND (Review_Completed_not_published OR Review_Completed_published):RS | 22/01/2018 | **2** |
| **Cochrane Reviews** |  |  |  |  |
| #1 | Search | ( ( propion* AND ( acidemia OR acidaemia OR aciduria ) ) ) OR ( ( ( ( propionyl AND ( coa OR "coenzyme a" ) ) OR propionic OR "PCCA" OR "PCCB" OR "PCC" ) AND ( deficien* OR disorder OR defect* OR insufficien* ) ) ) OR ( ( propionicaciduria OR propionicacidemia OR propionicacidaemia ) ) in Title, Abstract, Keywords | 22/01/2018 | **85** |
| **Rare disease organizations** | Orphanet | Site-specific search with relevant disease terms | 23/01/2018 | **3** |
|  | EIMD |  | 23/01/2018 | **1** |
|  | NORD |  | 23/01/2018 | **8** |
|  | CORD |  | 23/01/2018 | **0** |
|  | EURORDIS |  | 23/01/2018 | **0** |
|  | NIH |  | 23/01/2018 | **20** |
|  | GARD |  | 23/01/2018 | **0** |
|  | CLIMB |  | 23/01/2018 | **0** |
|  | OAUK |  | 23/01/2018 | **0** |
|  | OAA |  | 23/01/2018 | **42** |
|  | PA Foundation |  | 23/01/2018 | **13** |
|  | PARnet |  | 23/01/2018 | **8** |

*Supplementary Table 2 Exclusion criteria during the title and abstract screening*

| Irrelevant title without English abstract | All articles with irrelevant titles and without English abstract were excluded at this step. |
| --- | --- |
| Not related to PA | Since the literature databases cover a wide variety of scientific areas, studies that have no relevance to PA were excluded at this step. |
| No human subjects in the scope of the study | Studies not investigating human subjects were excluded at this step. |
| Editorial, letter, conference abstract or review | Editorials, letters, conference abstracts and non-systematic reviews were excluded at this criterion. All systematic literature reviews that seemed to be relevant for the epidemiology/natural disease history of PA were kept. |
| Case study or case series | Due to the rarity of the disease a minimum patient number was not defined, but if the abstract defined the paper as a case study or a description of a few case studies, yet did not publish any estimation of disease occurrence or aggregated results, then the study was excluded at this step. |
| No relevant data on epidemiology/natural disease history of PA | All studies that contained no relevant data on the epidemiology/natural disease history of PA were excluded at this last step. |

*Supplementary Table 3 Definitions of epidemiological measures*

| Measure | Definition |
| --- | --- |
| Birth prevalence | Number of cases with a birth defect in a defined area and time period divided by the number of live births in that area and time period. National aggregate statistics and newborn screening programs with a high sensitivity and specificity and ~100% population coverage are considered to be the most reliable sources to measure the birth prevalence in a population. |
| Estimated birth prevalence | The identified (and subsequently confirmed) cases during a period of newborn screening are divided by the number of screened newborns in the same period of time. A newborn screening program without ~100% coverage can only provide estimations on the true birth prevalence in the population. |
| Cumulative incidence in the birth cohort | The newly diagnosed cases within a specified birth cohort during a period of time are divided by the total number of subjects in the birth cohort. The observational period is equal to the period of birth cohort. |
| Lifetime risk at birth estimated by the diagnosis period method | The newly diagnosed cases in a period of time are divided by all live births in the same period of time. Lifetime risk is a special case of cumulative incidence in which the period of time studied is the entire remaining lifetime. The diagnosis (DX) method is calculated as the number of patients with a particular (or specific) diagnosis in the observational period divided by the number of live births during the same period [20]. |
| Point prevalence | Proportion of people in a population who have a disease or condition (proportion of diagnosed patients) at a particular time point. |
